# Control of Porosity and Pore Size of Metal Reinforced Carbon Nanotube Membranes

**DOI:** 10.3390/membranes1010025

**Published:** 2010-12-21

**Authors:** Ludovic Dumee, Leonora Velleman, Kallista Sears, Matthew Hill, Jurg Schutz, Niall Finn, Mikel Duke, Stephen Gray

**Affiliations:** 1 CSIRO Materials Science and Engineering, Bayview Avenue, Clayton, Victoria, 3168, Australia; E-Mails: kallista.sears@csiro.au (K.S.); matthew.hill@csiro.au (M.H.); jurg.schutz@csiro.au (J.S.); niall.finn@csiro.au (N.F.); 2 Institute for Sustainability and Innovation at Victoria University, PO Box 14428, Werribee, Victoria, 3030, Australia; E-Mails: mikel.duke@vu.edu.au (M.D.); Stephen.Gray@vu.edu.au (S.G.); 3 School of Chemical and Physical Sciences, Sturt road, Flinders University, Bedford Park, Adelaide, South Australia, 5042, Australia; E-Mail: Leonora.Velleman@flinders.edu.au

**Keywords:** carbon nanotube, nano-composite, gold, electroless plating, porosity

## Abstract

Membranes are crucial in modern industry and both new technologies and materials need to be designed to achieve higher selectivity and performance. Exotic materials such as nanoparticles offer promising perspectives, and combining both their very high specific surface area and the possibility to incorporate them into macrostructures have already shown to substantially increase the membrane performance. In this paper we report on the fabrication and engineering of metal-reinforced carbon nanotube (CNT) Bucky-Paper (BP) composites with tuneable porosity and surface pore size. A BP is an entangled mesh non-woven like structure of nanotubes. Pure CNT BPs present both very high porosity (>90%) and specific surface area (>400 m^2^/g). Furthermore, their pore size is generally between 20–50 nm making them promising candidates for various membrane and separation applications. Both electro-plating and electroless plating techniques were used to plate different series of BPs and offered various degrees of success. Here we will report mainly on electroless plated gold/CNT composites. The benefit of this method resides in the versatility of the plating and the opportunity to tune both average pore size and porosity of the structure with a high degree of reproducibility. The CNT BPs were first oxidized by short UV/O_3_ treatment, followed by successive immersion in different plating solutions. The morphology and properties of these samples has been investigated and their performance in air permeation and gas adsorption will be reported.

## 1. Introduction

Nano particles [1-3] and Carbon Nanotubes (CNT) [4] have attracted increasing interest over the past 20 years. Recently CNTs have been incorporated into composite structures and used as both porous and dense membranes for ultrafiltration [5], nanofiltration [6-8] and gas separation [9]. The inner diameter of CNTs have been used as pores for nanofiltration and for water desalination [10], while bucky-papers [11], or entangled meshes of CNTs, have been used for membrane distillation [12,13] and dye [14] and bacteria removal [15]. BPs offer naturally very high specific surface areas and their potential for gas permeation has been reported in the past [16,17] but better control of their porosity and surface chemistry could lead to higher gas adsorption and separation membranes. For example adding metal nanoparticles onto CNTs could lead to very sensitive gas sensors [18] and to higher specific surface area composites, presenting higher adsorption [19,20] or storage capacities [21,22]. Metal particles could also be surface treated to change the pore and adsorption properties. Previously, groups reported on the fabrication of copper/CNT composite [23] and on gold colloidal-CNT composite for sensing applications [24,25]. Furthermore, work has also been carried out to process pure gold nanotubes that can be surface treated to enhance the selectivity of their pores by adding thiols at their tips [26]. There are clearly many avenues for modifying the structures and chemistry of nanotubes for membrane separation applications and there is a need for controlled pore size and stable nano-structured that can resist into harsh pressure and temperature conditions.

In this paper we report on novel copper/CNT and gold/CNT composites where metal was grown into a BP structure by electrochemical processes. Both electroplating and electroless deposition were performed and compare to tune both the average pore size and the porosity of the BPs. Inner properties of the BP were such as their specific surface area and porosity were investigated. Changes induced by the gold on the BP electrical conductivities were also monitored while their permeation to air was measured.

## 2. Experimental Section

### 2.1. Carbon Nanotube Stock and Bucky-paper (BP) Fabrication

CNTs were grown by chemical vapor deposition at the CSIRO Materials Science and Engineering department by method reported elsewhere [27]. A 1–5 nm thick iron catalyst film was deposited onto a silicon substrate bearing a thin Silicon Dioxide layer. A mixture of Helium (95%) and Acetylene (5%) was used as the carbon feedstock and heated to between 650 and 750 °C. The CNTs typically have an outer diameter of ∼10–15 nm and length of 150–300 μm. Those multi walled CNTs typically present 8 to 10 walls in their structure. CNTs were naturally grown as a forest on their silicon wafer support and were scraped from this support prior to further treatments [28]. The CNT BP membranes were processed by vacuum filtration of CNTs dispersed in 99.8% pure propan-2-ol [29-31]. Well dispersed CNT suspensions were obtained by repeating 5 cycles of sonication for 10 min intervals at a power of 150 W and freezing at −17 °C. Vacuum filtration was performed with a 47 mm diameter Millipore filtration unit with house line vacuum (dP = −95 kPa). The CNTs were filtered onto a poly (ether sulphone) (PES) 0.2 μm-pore size Millipore membrane and then pealed off to form a self-supporting membrane [11,12,29-32].

### 2.2. UV/Ozone Treatment of the Nanotube Surface

To activate the surface of the nanotubes, UV/ozone treatment was performed on the nanotube by placing the BP into a flow of UV induced ozone. Ozone attacked the CNT surface and formed hydroxyl and carboxylic groups as well as surface defects [33]. This step was critical to enhance the wettability of the membranes by the processing solutions during the plating steps. The CNT BPs were exposed for 10 min to a UV lamp in a flow of oxygen at 5 cm distance. This treatment was used to create functional groups on the nanotubes to act as anchoring points for the subsequent plating procedure [34].

### 2.3. Electro Plating Procedure

Electroplating (EP) of copper was first performed on both as grown and UV/ozone treated CNT BPs. The samples were first wet for 2 h in a mixture of deionised water and ethanol 80:20 followed by electroplating at −1 V in a 100 g/L solution of copper sulphate. The treatment time varied from 2 min up to 40 min. The CNTs acted as the working electrode while an Ag/AgCl electrode acted as reference and a platform wire as a counter.

### 2.4. Electroless Plating Procedure

Electroless (ES) deposition was performed to fabricate gold reinforced BPs. The procedure for electroless gold deposition within porous materials has been previously described by Martin *et al.* [35]. In the first step referred to as sensitization, the membrane was immersed in a solution of 0.026 M SnCl_2_ and 0.07 M trifluoroacetic acid in a solvent of 50:50 methanol:water for 45 min followed by rinsing in methanol for 5 min. This was followed by the second step, referred to as activation, where the membrane was immersed in a solution of 0.029 M ammoniacal AgNO_3_ for 30 min. The membrane was then rinsed in methanol for 5 min and immersed in water before placing the membranes in the plating bath. In the third step, referred to as displacement deposition, the silver coated membrane was immersed in the gold plating solution consisting of 0.079 M Na_3_Au(SO_3_)_2_, 0.127 M Na_2_SO_3_, 0.625 M formaldehyde and 0.025 M NaHCO_3_. The temperature of this bath was ∼1−4 °C with pH = 8. A plating time of 20 h was used in this study which significantly reduces the pore size of the membrane without closing the pores. Finally, the membranes were thoroughly rinsed in deionised water and ethanol and air-dried at room temperature.

### 2.5. Characterisation of the Composite Membranes

Several characterisation techniques were carried out on the CNTs before and after gold plating. Determination of the pore size distribution and average pore size was estimated by SEM image analysis performed on a Philips FEG SEM at 2 keV [13,36,37]. Porosity measurements were carried out on an AccuPyc II 1340 1 cm^3^ Gas Displacement Pycnometer from Micromeritics at 19 Psi. The gold content in the structure was determined by Thermo Gravimetric Analysis (TGA) (Perkin Elmer, TGA 7) analysis using a non treated CNT BP as the reference. Tests were carried out at a rate of 10 °C/min and up to 900 °C. Impedance measurements were also done to evaluate the impact of the gold addition on the electrical conductivity of the samples. The BPs were cut in thin strips of 30 mm by 2 mm and their resistance measured with a multi-meter. Raw and untreated nanotubes were used as benchmarks to compare with the functionalised and plated CNTs.

Gas permeation measurements were performed by placing the membrane in an o-ring sealed holder which separates the large feed volume from a much smaller permeate vessel. After loading the membrane, both the feed and permeate vessels were evacuated. The feed vessel was then isolated from both the vacuum and membrane holder and filled to atmospheric pressure with high purity gas. The permeate was then isolated from vacuum and the pressure rise monitored over time until equilibrium was reached.

**Figure 1:**
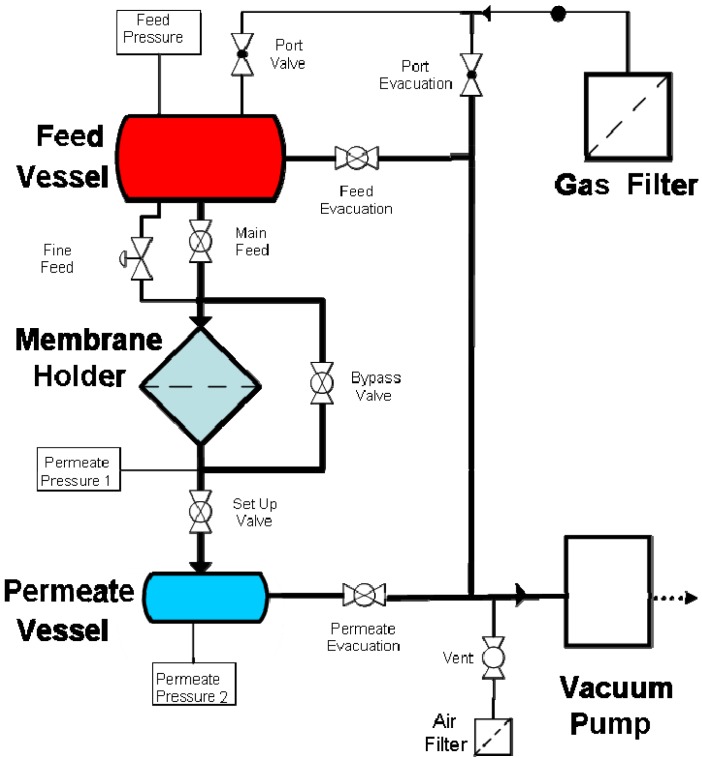
Gas rig configuration.

Permeation tests of filtered and dried air undertaken to evaluate the impact of the change in porosity and pore size on the permeation properties. A schematic of the rig is given in Figure 1.

## 3. Results and Discussion

### 3.1. Morphology of the Nanotubes after UV/Ozone Treatment

**Figure 2:**
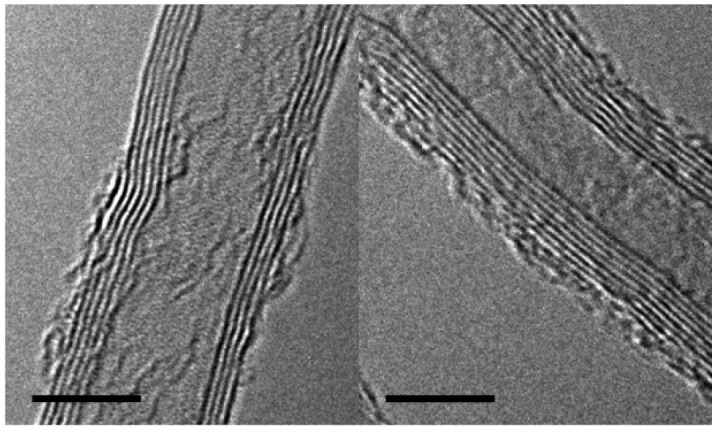
TEM images of the CNTs before (left) and after (right) UV/ozone treatment. Scale bar corresponds to a length of 5 nm. Damage on the outer walls is visible after UV/ozone exposure.

The initial CNT density was found to be close to ∼1,010 by analyzing silicon growth wafers after removing the CNTs. The sites of growth were visible and hand counted on a number of samples. The CNTs were damaged by the ozone treatment. The outer walls of the CNTs showed either partial vacancies or breakages over the length of the samples examined by TEM. Examples of the defects are shown in Figure 2. Some of those defects are hosts to hydroxyl or carboxylic groups while others are recombined carbons. Amorphous carbon is also visible on the outer walls. The addition of those functional groups was not found to alter neither the geometrical (pore size, porosity) nor the permeation properties of the membrane.

### 3.2. Morphology of the Samples

**Figure 3:**
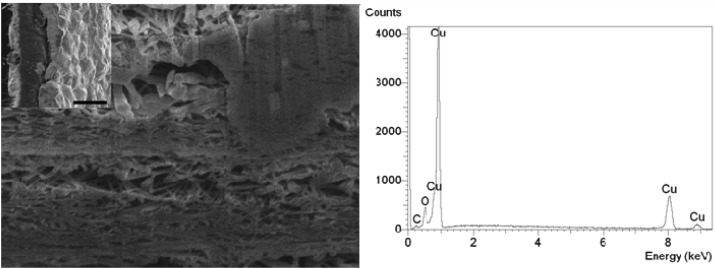
Focus ion beamed cross section of a copper plated BP. Left image (insert) shows that copper is present on the surface, while the main SEM image shows that aggregates of copper are being formed within the pores of the structure as demonstrated by the EDS analysis. The scale bars respectively correspond to 10 μm for the insert and 1 μm for the main image.

CNT BPs are typically randomly distributed mats of nanotubes. The morphology of the electroplated (EP) samples was different from that of the electroless (ES) plated ones. Those differences are inherent to the processing conditions and can be attributed to the way the metals are being attracted and deposited onto the surface. Furthermore, it is also likely that the wetting of the inner BP by the process liquids is critical to achieve homogeneous deposition. The rate of deposition of metals onto the EP coated BPs is dependent on the applied voltage. For ES coated BPs the coverage of gold is dependent of the number of sites available for the chelation of tin originating from the initial ozone treatment while the deposition rate is dependent on gold plating bath conditions such as the temperature, concentration and pH. These factors all impact the resulting metal-BP structure. The EP process created both a dense metallic layer at the surface of the electrode and randomly local aggregates blobs of metals within the pores of the BP, as shown in Figure 3 on both SEM cross section and EDS graph. The EDS was done on the top right hand side of the image where a semi dense material is partially screening the CNTs. The aggregate size did vary and they were mostly localised close to the membrane surface. The dispersion of the metal through the BP proved non-homogeneous resulting in poor reproducibility and inadequate determination of the average pore size and shape.

**Figure 4:**
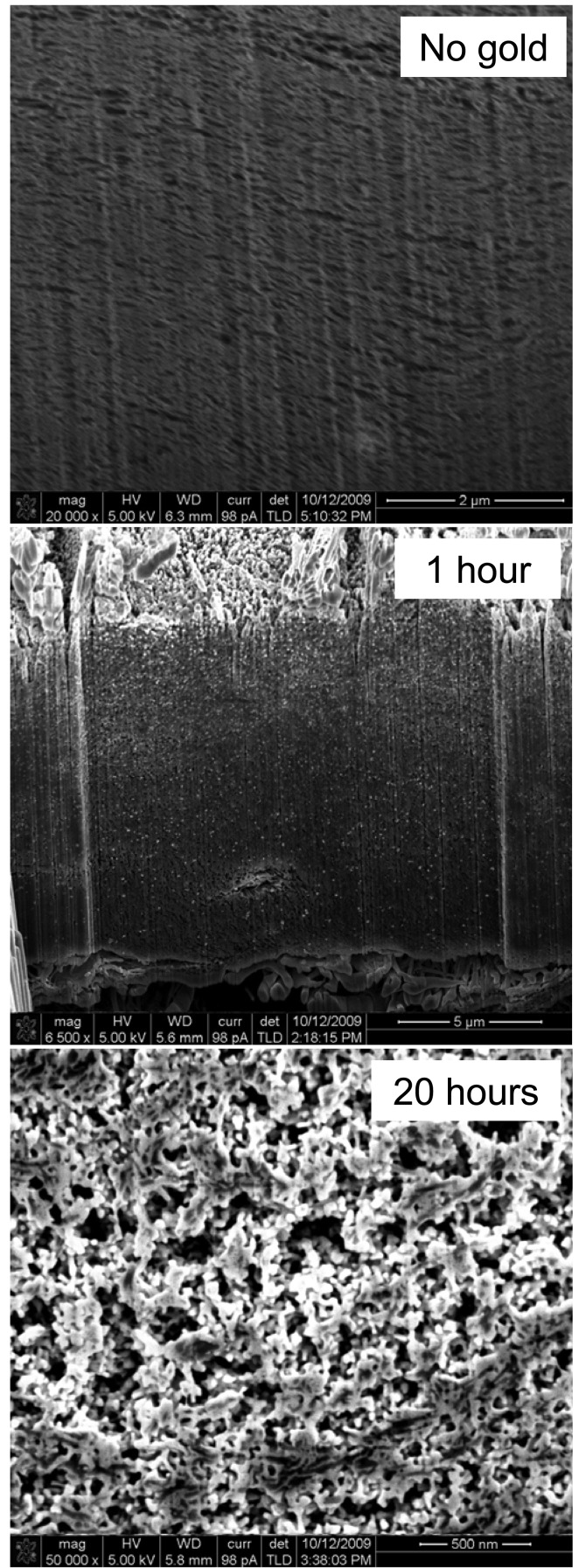
Scanning Electron Micrographs taken on a Focus Ion Beam (FIB) SEM. Cross sections were performed at an intensity of 7 nA and images taken at a tilt angle of 53°. The working distance was 7.5 mm and the surface was not coated with any conducting metal.

For these reasons ES was carried out on BPs fabricated from the same kind of nanotubes. The ES process proved to be more suitable to produce nice homogeneous structures, as shown in Figure 4. Gold was first deposited on defects created on the CNTs. From those sites it grew and covered the CNTs progressively but without filling up the structure or blocking the pores. Figure 4 clearly shows that the structure stays porous even after lengthy plating times. Particles of gold first deposited on the nanotubes were tin and silver adsorbed. Their size was found to be close to the CNT diameter for small plating times. Once coating spread on the nanotubes aggregates of ∼10–30 nm were found around the CNTs after 1 h. After approximately 10 h of plating a continuous network was being formed inside the BP on the CNTs leading to a new homogenous surface made of pure gold in which the CNTs were entrapped.

The gold content as a percentage of the total sample mass in the membranes was determined by Thermo Gravimetric Analysis (TGA) in nitrogen. CNTs were shown to be stable at low temperature and start decomposing around 500–550 °C. Residuals, such as possible iron catalysts or other inorganic particles after carbonisation were found to be negligible compared to the carbon mass on our reference samples. In the case of the gold plated samples, the CNTs started decomposing at lower temperatures in the range of 420 °C to 590 °C. This was attributed to the presence of the hydroxyl groups and defects created during the ozone treatment which left free sites for attack by oxygen.

The gold content rose from ∼10% for 1 h of plating up to 90% after 26 h. The slope of the coating rate decreases after 20 h and seems to reach a plateau (Table 1) which is attributed to the progressive decrease in gold content in the BP pores. It has been shown that the deposition of electroless gold is preferentially deposited on the surface of the membrane rather than within the confines of a pore, therefore once the pore size becomes restricted gold deposition will not be favoured within the BP and hence the deposition rate of gold within the membrane will decrease [38]. Furthermore the plating was performed in static mode, meaning that the BP were immersed and kept in the solution but no flow was forced through their pores once the process started. To sustain such a high rate, one would need to inject fresh solution in situ during the test or ensure better flow and homogeneity of the solution across the BP. This increase in gold content also fits well with the decrease in linear resistivity of the BP samples. Table 1 presents the impact of the plating time on the resistance of strips of BP. The sample resistance sharply decreased even after the addition of small amounts of gold.

**Table 1:** Properties of the gold electroless plated samples.

Plating time	h	0	5	20	30
Gold content	%	0	28	89	90
Porosity	%	90	78	62	41
Surface pore size	nm	25	23.5	15.8	7
Resistance	Ohm/cm	924	1.46	0.1	0.02
Specific surface area	m^2^/g	197	229	88	37

Furthermore the internal pores were still available for Helium and N_2_ as both pycnometer and BET tests indicated that gas could still penetrate the membranes. The BET surface area increased during the first hours of treatment, which is linked to the presence of a low amount of well dispersed and growing gold nanoparticles across the sample (Table 1). The gold nanoparticles have a very high specific surface area which adds to the already large CNT specific surface area. In fact, as the plating continues the nanoparticles grow and coalesce which results in a reduction of the specific surface area of the sample after 5 h of treatment. At longer plating times (above 10 h) the gold begins to fill up the pores thus reducing the BET surface area to less than the original BP sample. This trend can also be correlated to the electrical resistance of the samples. Even with 1 h of plating the resistance is decreased by nearly 3 decades showing that even small amounts of gold can have major impacts on the CNT BP properties and on their potential application. The porosity measurements correlated very well with the trend obtained from the SEM images. Pycnometer tests showed that (Table 1) the porosity decreases from 90% for a pure CNT BP down to 40% within the first 30 hours of plating. The average pore size was determined by analysing surface images of the samples (Table 1) in a method described elsewhere. The average pore size decreased from 25 nm for pure CNT BPs down to less then 7 nm making the stiff and yet flexible membranes promising candidates for ultrafiltration. Longer plating time could also lead to smaller surface pores and possibly a use for nanofiltration and gas separation.

### 3.3. Gas Permeation and Adsorption

**Figure 5:**
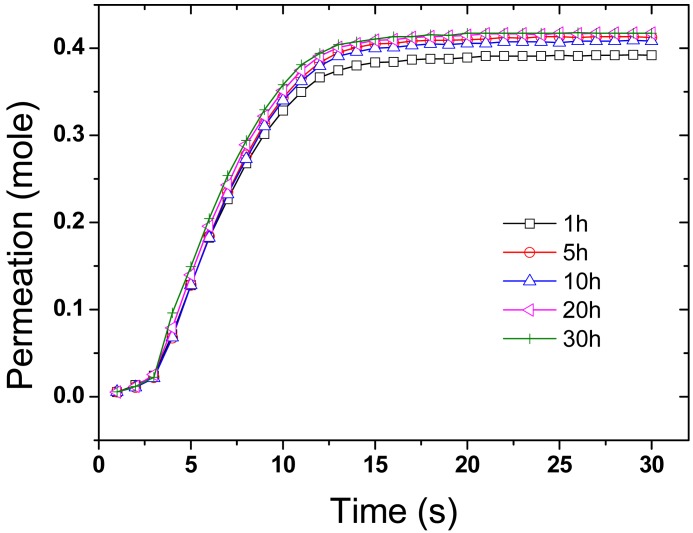
Dried air permeation across gold plated CNT BP. The corresponding plating times are given in hours in the legend.

Gas permeation tests with dried air were performed on a series of membranes. The tests were carried out in a closed system where the feed was filtered, and dried, air and the permeate evacuated to low pressure. Measurements were conducted by passing dried air pass through the membrane from the feed to the permeate side until equilibrium is reached (Figure 5). No significant changes in permeation were observed at the different plating times and an average rate of permeation of 0.6 mol·s^−1^ (+/−0.05) was calculated from the curves thus indicating that the change in pore size had no major significance on the permeation.

## 4. Conclusions

BPs were successfully templated with gold nanoparticles to produce denser and less porous membranes. We demonstrated that even after 30 hours of plating they remained porous and no significant change in air permeation was noticeable. The change in resistance of the strips is directly correlated to the amount of gold in the structure and this could be used for the design of improved sensors for toxic metals of organic compound detection. The very high CO_2_ adsorption on the GOLD/CNT composites is explained by the affinity and activation of CO_2_ by the gold surface. Electroless plating reveals to be an interesting method for plating nano-porous materials and expose metal nanoparticles offering catalytic or activation properties.

A range of molecules can be attached to the gold via a thiol linkage, thereby modifying the membrane surfaces and pores with functional groups. The selective transport of permeate molecules can be controlled and enhanced with the immobilization of appropriate functional groups on the gold. Furthermore longer plating times may lead to denser membranes with tighter pores applicable for nanofiltration and potentially for separation of organic gases.

## References

[1] Cooper C.H., Cummings A.G., Starrostin M.Y., Honsinger C.P. (2007). Purification of Fluids with Nanomaterials.

[2] Shao L., Samseth J., Hagg M.-B. (2009). Crosslinking and stabilization of nanoparticle filled PMP nanocomposite membranes for gas separations. J. Membr. Sci..

[3] Ahn J., Chung W.-J., Pinnau I., Guiver M.D. (2008). Polysulfone/silica nanoparticle mixed-matrix membranes for gas separation. J. Membr. Sci..

[4] Iijima S. (1991). Helical microtubules of graphitic carbon. Nature.

[5] Qiu S., Wu L., Pan X., Zhang L., Chen H., Gao C. (2009). Preparation and properties of functionalized carbon nanotube/PSF blend ultrafiltration membranes. J. Membr. Sci..

[6] Holt J. (2006). Fast transport through carbon nanotubes and implications for water treatment. Innovative Technical Solutions for Water Management in Australia.

[7] Holt J.K., Noy A., Huser T., Eaglesham D., Bakajin O. (2004). Fabrication of a carbon nanotube-embedded silicon nitride membrane for studies of nanometer-scale mass transport. Nano Lett..

[8] Hinds B.J., Chopra N., Rantell T., Andrews R., Gavalas V., Bachas L.G. (2004). Aligned multiwalled carbon nanotube membranes. Science.

[9] Cong H., Zhang J., Radosz M., Shen Y. (2007). Carbon nanotube composite membranes of brominated poly (2,6-diphenyl-1,4-phenylene oxide) for gas separation. J. Membr. Sci..

[10] Fornasiero F., Park H.G., Holt J.K., Stadermann M., Grigoropoulos C.P., Noy A., Bakajin O. (2008). Ion exclusion by sub-2-nm carbon nanotube pores. PNAS.

[11] Gou J., Liang Z.Y., Wang B. (2004). Experimental design and optimization of dispersion process for single-walled carbon nanotube bucky paper. Int. J. Nanosci..

[12] Dumee L., Sears K., Schutz J., Finn N., Duke M., Gray S. Design and characterisation of carbon nanotube Bucky-Paper membranes for membrane distillation.

[13] Dumee L.F., Sears K., Schutz J., Finn N., Huynh C., Hawkins S., Duke M., Gray S. (2010). Characterization and evaluation of carbon nanotube Bucky-Paper membranes for direct contact membrane distillation. J. Membr. Sci..

[14] Gong J.-L., Wang B., Zeng G.-M., Yang C.-P., Niu C.-G., Niu Q.-Y., Zhou W.-J., Liang Y. (2009). Removal of cationic dyes from aqueous solution using magnetic multi-wall carbon nanotube nanocomposite as adsorbent. J. Hazard. Mater..

[15] Brady-Esetvez A.S., Kang S., Elimelech M. (2008). A single-walled-carbon-nanotube filter for removal of viral and bacterial pathogens. Small.

[16] Smajda R., Kukovecz A., Konya Z., Kiricsi I. (2007). Structure and gas permeability of multi-wall carbon nanotube buckypapers. Carbon.

[17] Cooper S.M., Chuang H.F., Cinke M., Cruden B.A., Meyyappan M. (2003). Gas permeability of a buckypaper membrane. Nano Lett..

[18] Fam D.W.H., Tok A.I.Y., Palaniappan A., Nopphawan P., Lohani A., Mhaisalkar S.G. (2009). Selective sensing of hydrogen sulphide using silver nanoparticle decorated carbon nanotubes. Sensor. Actuator. B Chem..

[19] Reddy A., Mohana L., Ramaprabhu S. (2008). Hydrogen adsorption properties of single-walled carbon nanotube—Nanocrystalline platinum composites. Int. J. Hydrogen Energ..

[20] Lin K.-Y., Tsai W.-T., Yang T.-J. Effect of Ni nanoparticle distribution on hydrogen uptake in carbon nanotubes. J. Power Sourc..

[21] Chen C.-Y., Lin K.-Y., Tsai W.-T., Chang J.-K., Tseng C.-M. (2010). Electroless deposition of Ni nanoparticles on carbon nanotubes with the aid of supercritical CO_2_ fluid and a synergistic hydrogen storage property of the composite. Int. J. Hydrogen Energ..

[22] Zlotea C., Campesi R., Cuevas F., Leroy E., Dibandjo P., Volkringer C., Loiseau T., Ferey G., Latroche M. (2010). Pd nanoparticles embedded into a metal-organic framework: Synthesis, structural characteristics, and hydrogen sorption properties. J. Am. Chem. Soc..

[23] Yuan D., Liu Y. (2006). Electroless deposition of Cu on multiwalled carbon nanotubes. Rare Metal..

[24] Kim J., Rabbani M.M., Kim D., Ree M., Yeum J.H., Ko C.H., Kim Y., Bae J.-S., Oh W. (2010). Structural and electrochemical properties of gold-deposited carbon nanotube composites. Curr. Appl. Phys..

[25] Manso J., Mena M.L., Yanez-Sedeno P., Pingarron J. (2007). Electrochemical biosensors based on colloidal gold-carbon nanotubes composite electrodes. J. Electroanal. Chem..

[26] Velleman L., Shapter J.G., Losic D. (2009). Gold nanotube membranes functionalised with fluorinated thiols for selective molecular transport. J. Membr. Sci..

[27] Kumar M., Ando Y. (2010). Chemical vapor deposition of carbon nanotubes: A review on growth mechanism and mass production. JNN.

[28] Huynh C.P., Hawkins S.C. (2010). Understanding the synthesis of directly spinnable carbon nanotube forests. Carbon.

[29] Gou J. (2006). Single-walled nanotube bucky paper and nanocomposite. Polymer Int..

[30] Kim B.Y.A., Muramatsu H., Hayashi T., Endo M., Terrones M., Dresselhaus M.S. (2006). Fabrication of high purity, double-walled carbon nanotube buckypaper. Chem. Vap. Depos..

[31] Muramatsu H., Hayashi T., Kim Y.A., Shimamoto D., Kim Y.J., Tantrakarn K., Endo M., Terrones M., Dresselhaus M.S. (2005). Pore structure and oxidation stability of double-walled carbon nanotube-derived bucky paper. Chem. Phys. Lett..

[32] Dumee L.F., Sears K., Schütz J., Finn N., Huynh C., Hawkins S., Duke M., Gray S. (2010). Characterization and evaluation of carbon nanotube bucky-paper membranes for direct contact membrane distillation. J. Membr. Sci..

[33] Agrawal S., Raghuveer M.S., Li H., Ramanath G. (2007). Defect-induced electrical conductivity increase in individual multiwalled carbon nanotubes. Appl. Phys. Lett..

[34] Sham M.-L., Kim J.-K. (2006). Surface functionalities of multi-wall carbon nanotubes after UV/Ozone and TETA treatments. Carbon.

[35] Menon V.P., Martin C.R. (1995). Fabrication and evaluation of nanoelectrode ensembles. Anal. Chem..

[36] Sears K., Dumee L., Schütz J., M., Huynh C., Hawkins S., Duke M., Gray S. (2009). Recent developments in carbon nanotube membranes for water purification and gas separation. Materials.

[37] Bessieres A., Meireles M., Coratger R., Beauvillain J., Sanchez V. (1996). Investigations of surface properties of polymeric membranes by near field microscopy. J. Membr. Sci..

[38] Jirage K.B., Hulteen J.C., Martin C.R. (1997). Nanotubule-based molecular-filtration membranes. Science.

[39] Browne V.M., Carley A.F., Copperthwaite R.G., Davies P.R., Moser E.M., Roberts M.W. (1991). Activation of carbon dioxide at bismuth, gold and copper surfaces. Appl. Surf. Sci..

